# Mobility and posterior tibial tendon dysfunction (PTTD) in persons with flat foot: a case control study

**DOI:** 10.1186/1757-1146-8-S2-O16

**Published:** 2015-09-22

**Authors:** Marabelle L Heng, Pui W Kong

**Affiliations:** 1 Podiatry Department, Singapore General Hospital, Singapore 169608; 2 Physical Education & Sports Science Academic Group, National Institute of Education, Nanyang Technological University, Singapore 637616

**Keywords:** Biomechanical etiology, hypermobility, stiffness, first ray, metatarsophalangeal joint

## Background

Posterior tibial tendon dysfunction (PTTD) is a progressive and debilitating condition. Flat foot is a known associated factor of PTTD but there are still many questions about the biomechanical etiology of PTTD. Hypermobility in the first ray and first metatarsophalangeal joint (MPJ) is thought to be a causative factor in various foot pathologies including PTTD, however the association is not well established. This study aimed to (i) investigate whether hypermobility was associated with development of PTTD in flat footed persons and (ii) report a novel technique of quantifying the stiffness of foot joints.

## Methods

Sixteen flat footed subjects exhibiting PTTD symptoms were compared with 16 asymptomatic flat footed controls matched for age, gender and body mass index. First ray and first MPJ joint stiffness were quantified as such: (a) force required to displace the joint was recorded using a finger sleeve pressure pad; (b1) joint displacement for first ray (mm) was measured using a simple ruler device while (b2) joint angular displacement for first MPJ (^o^) was determined from video analysis; (c) a force by displacement graph of 10 trials was plotted and gradient of the best line of fit gave the stiffness value. In addition, Beighton score was used to assess generalised joint mobility.

## Results

There were no significant differences in first ray stiffness [PTTD: 0.78(0.59) N/mm, control: 0.88(0.66) N/mm; p = .73] or first MPJ stiffness [PTTD: 11.7 (0.7) Nmm/^o^, control: 11.0(5.0) Nmm/^o^; p = .85] between the PTTD and control groups. Similarly, no difference in Beighton score was noted [PTTD: 3.13(2.25); control: 2.88(2.36); p = .61].

## Conclusions

Foot joint mobility was not associated with PTTD in flat footed individuals. Further studies are required to confirm the biomechanical etiology of PTTD. The novel technique used in this study displayed potential to be further developed for clinical use.

